# PyVADesign: a python-based cloning tool for one-step generation of large mutant libraries

**DOI:** 10.1093/bioinformatics/btaf433

**Published:** 2025-09-01

**Authors:** R C M Kuin, M H Lamers, G J P van Westen

**Affiliations:** Computational Drug Discovery, Medicinal Chemistry, Leiden University, Leiden, 2333 CC, The Netherlands; Department of Cell and Chemical Biology, Leiden University Medical Center, Leiden, 2333 ZA, The Netherlands; Department of Cell and Chemical Biology, Leiden University Medical Center, Leiden, 2333 ZA, The Netherlands; Computational Drug Discovery, Medicinal Chemistry, Leiden University, Leiden, 2333 CC, The Netherlands

## Abstract

**Motivation:**

The generation and analysis of diverse mutants of a protein is a powerful tool for understanding protein function. However, generating such mutants can be time-consuming, while the commercial option of buying a series of mutant plasmids can be expensive. In contrast, the insertion of a synthesized double-stranded DNA (dsDNA) fragment into a plasmid is a fast and low-cost method to generate a large library of mutants with one or more point mutations, insertions, or deletions.

**Results:**

To aid in the design of these DNA fragments, we have developed PyVADesign: a Python package that makes the design and ordering of dsDNA fragments straightforward and cost-effective. In PyVADesign, the mutations of interest are clustered in different cloning groups for efficient exchange into the target plasmid. Additionally, primers that prepare the target plasmid for insertion of the dsDNA fragment, as well as primers for sequencing, are automatically designed within the same program.

**Availability and implementation:**

PyVADesign is open source and available at https://github.com/CDDLeiden/PyVADesign and archived via Zenodo (https://doi.org/10.5281/zenodo.15057525).

## 1 Introduction

Proteins are essential to all biological systems due to their diverse functional roles, ranging from catalyzing biochemical reactions to regulating cellular processes and enabling communication between cells (Agarwal, 2006, Westermarck *et al.* 2013, Morris *et al.* 2022). Mutations in proteins can lead to altered functions, which can be detrimental or, in some cases, provide evolutionary advantages, thus playing a crucial role in both disease mechanisms and adaptive evolution (Loewe and Hill, 2010). Consequently, studying the behavior of mutants provides valuable insights into protein function, aids in protein engineering, helps explain disease development, and elucidates drug resistance (Tait, 1999, Sen *et al.* 2022, Sellés Vidal *et al.* 2023). Although generating mutants can be time-consuming, it remains a fundamental aspect of molecular biology (Forloni *et al.* 2019).

Mutations are traditionally created through site-directed mutagenesis by PCR (Weiner *et al.* 1994, Bachman, 2013). As all mutants are introduced on a per-case basis, creating different mutations can become time-consuming, as each mutation demands a unique set of primers and PCR reaction, which becomes labor-intensive for larger sets of mutants. In addition, the generation of multiple mutations in a gene using a single PCR is limited to residues that are close to each other in the sequence. For multiple mutations that are spaced further apart, there is no other option than to go through multiple rounds of mutagenesis. Over recent years, bioinformatics tools have been developed to design oligo’s automatically for mutagenesis and gene assembly, reducing experimental planning (Untergasser *et al.* 2012, Hiraga *et al.* 2021, Leonte *et al.* 2025). To further decrease the experimental workload, synthesis of whole genes or plasmids offers a fast alternative but can become expensive when multiple mutant versions are required.

**Figure 1.**
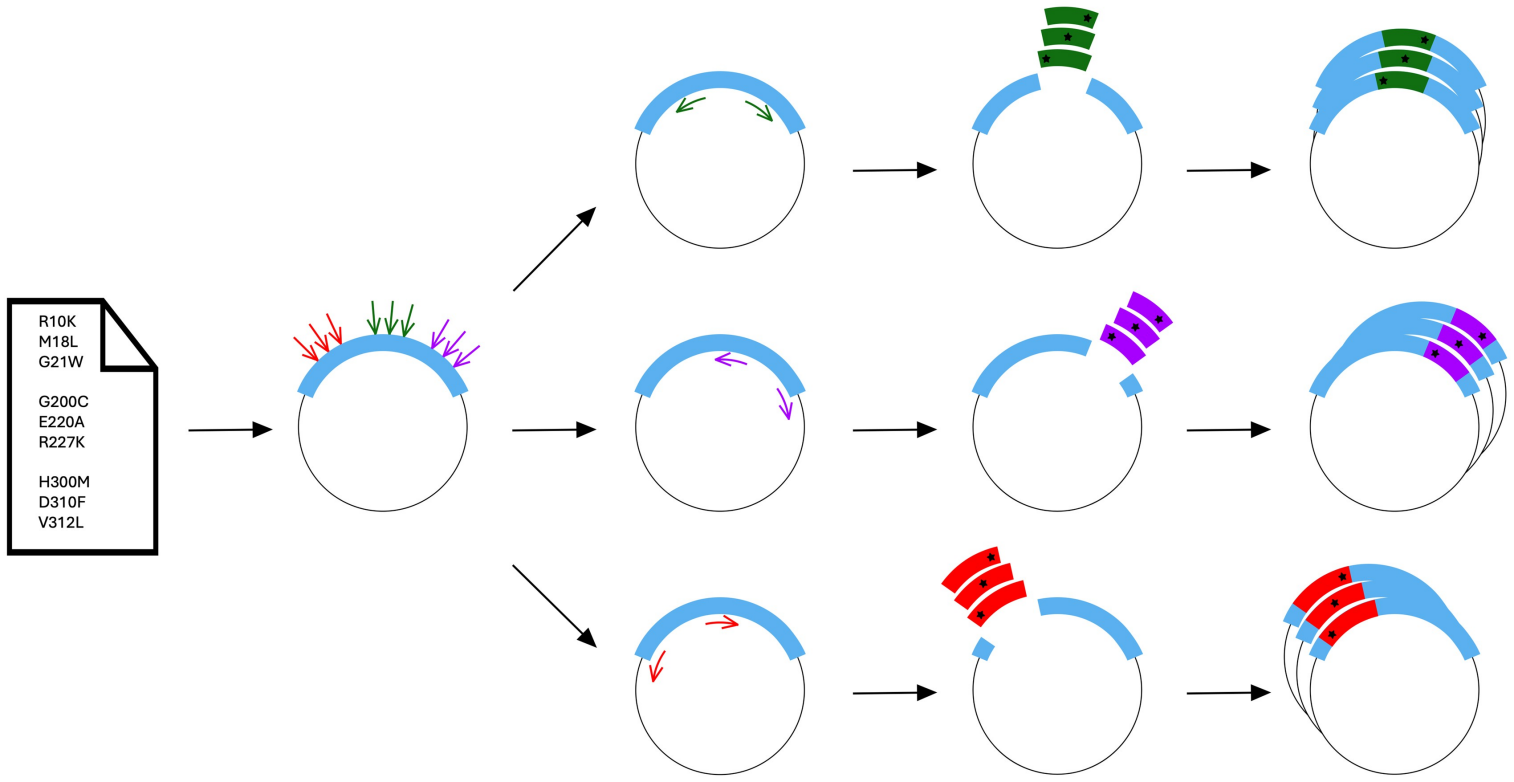
Graphical representation of the PyVADesign workflow. This workflow places each single mutation, or a group of mutations located within ∼200 bp, within a single dsDNA fragment, allowing for a rapid, parallel creation of multiple plasmids, without the need of sequential rounds of mutagenesis. The user’s input includes a plasmid sequence containing the gene of interest (blue) and a list of desired mutations. PyVADesign clusters the mutations on the gene of interest without explicit user input. Next, multiple dsDNA fragments are designed that each carry one or more mutations. In addition, primers are designed to linearize the target plasmid by PCR for insertion of the dsDNA fragment (shown in green, purple, and red colors). Finally, the dsDNA fragments are inserted into the linearized plasmids, creating a library of mutant plasmids in a single parallel step.

Alternatively, synthesis of short double-stranded DNA (dsDNA) fragments ranging from a few hundred to thousands of base pairs (bp), combined with easy-to-use cloning methods such as in vivo assembly (IVA) (García-Nafría *et al.* 2016), offers a quick and economic approach to generate large mutant libraries with one or more point mutations, as well as insertions or deletions (Hughes and Ellington, 2017). In addition to already existing tools, here we shorten the experimental workflow by enabling insertion of different dsDNA fragments in a single PCR-opened plasmid, each carrying one or more different mutations, thereby allowing multi-site-directed mutagenesis in a single step, even for paired mutations over 200 bp apart, without the need of sequential cloning steps. To streamline this process, we created PyVADesign, a Python package that makes the design and ordering of dsDNA fragments straightforward. Using PyVADesign, mutations of interest are clustered within the target gene, and a dsDNA fragment is designed for each variant; see Fig. 1. The program also automatically generates primers to prepare the expression plasmid for dsDNA fragment insertion, as well as primers for validation by DNA sequencing. The tool is open-source and user-friendly for scientists of all backgrounds.

## 2 Methods

### 2.1 dsDNA fragment design process

First, the desired mutations are grouped together into clusters using K-medoids clustering (Kaufman and Rousseau, 1990) in scikit-learn-extra (The scikit-learn-extra development team, 2020). Clustering is needed to find groups of input mutations that are close together in the DNA sequence. This allows for fitting multiple mutations in a single fragment region and subsequent easy single-step insertion into the target plasmid. Starting with one cluster, we assess if the resulting cluster sizes meet the size requirements. This process is repeated with an increasing number of clusters until the cluster sizes become smaller than the minimum size of the dsDNA fragment. The acceptable size range for dsDNA fragments varies depending on the synthesis provider but typically spans from approximately 150 to 5000 bp. Using this approach, fragment regions are created that cover a maximum of mutations and leave out regions for which no mutations are designed (Fig. 1, available as supplementary data at Bioinformatics online). For two or more simultaneous mutations, constraints are generated for paired data points, modifying the distance metric by reducing the distance between them to promote their co-clustering. The cluster selection depends on user preference and can be based on either fragment quantity or fragment length. The quantity-optimization option selects a clustering based on the number of different fragment regions, aiming to reduce the number of required PCRs. The length-optimized option prioritizes minimizing the total number of base pairs per region, which results in a higher number of smaller fragment regions compared to the quantity option, which are cheaper than the larger fragments.

After clustering, the mutations are assigned to a fragment region, and a dsDNA fragment is created for each single mutant or group of mutations that lie within one fragment region. For each mutation, the most frequently used codon in the selected (expression) organism is determined by retrieving the genome from the NCBI Entrez database (Sayers *et al.* 2022) and calculating the relative frequencies using Biotite (Kunzmann and Hamacher, 2018). Flanking sequences of at least 15 bp in length with a T_m_ between 47°C and 52°C are added to both ends of the dsDNA fragment that are required for the insertion into the target plasmid via IVA cloning (García-Nafría *et al.* 2016). Lastly, two silent mutations are included on both ends of the dsDNA fragment as an easy identification tool, bypassing the need to sequence the entire insert.

### 2.2 Primer design

After the dsDNA fragment design, two sets of primers are designed: one set of primers to open up the target plasmid to allow for insertion of the dsDNA fragment and one set of primers for sequencing of the created plasmid. Primers are designed using the Primer3 package (Rozen and Skaletsky, 2000, Untergasser *et al.* 2012). Settings for both primer sets can be adapted to user preferences through a settings file, allowing for customization of parameters such as optimal primer length, melting temperature, and GC content. Within the Primer3 package, the primers are also checked for the formation of secondary structure and off-target binding sites.

### 2.3 DNA synthesis

All dsDNA fragments and primers were ordered from IDT^TM^ (Leuven, Belgium). The dsDNA fragments were delivered in a 96-well plate at 10 ng/µL in nuclease-free water.

### 2.4 Preparation of target plasmid

The target plasmid was opened up using the designed primer set through PCR. PCR was performed by using KOD Hot Start Master Mix (Sigma-Aldrich), according to the manufacturer’s instructions. Briefly, each reaction contained 10 ng of plasmid, 0.3 μM of each primer, and 25 μL of KOD Hot Start Master Mix in a total reaction volume of 50 μL. PCR consisted of 20 cycles of denaturation at 95°C, annealing at 65°C, and extension at 72°C, with a final extension step of 225 seconds at 72°C. Reaction mixtures were treated with DpnI for 60 minutes to remove the parent plasmid. The linear PCR fragments were purified from a 1% agarose gel using the Wizard^®^ SV Gel and PCR Clean-Up System (Promega) and stored at −20°C until use.

### 2.5 Assembly of mutant plasmid

To assemble the mutant plasmid, 20 ng of the synthetic dsDNA fragment and 100 ng of the opened-up target plasmid were combined and transformed into *E. coli* DH5α cells using a heat shock method (Froger and Hall, 2007). Single colonies were picked and grown in 5 ml Luria-Bertani (LB) at 37°C overnight. DNA was extracted using the QIAprep Spin Miniprep Kit (Qiagen). All plasmids were sequenced by Illumina sequencing.

## 3 Results

The software package is written in Python version 3.9 and is organized into modules, each handling a separate task. If no customization is required, the package offers a command-line interface for quick setup of experiments with default settings, so the package is easy to use for molecular biologists. For more complex experimental designs, a settings file can be supplied to customize both the dsDNA fragment design process and primer parameters. Both the dsDNA design process and primer design process are also described in a step-by-step tutorial in the GitHub repository. The tool has been tested on Windows and Unix-based systems to ensure cross-platform functionality.

### 3.1 User input and considerations

To successfully run the design process, a text file with the mutations of interest is required, listing various types of mutations, including single-point mutations, multiple-point mutations, insertions, and deletions; see the Supporting Data for an example file, available as supplementary data at Bioinformatics online. It is important to note that, depending on the sizes of the dsDNA fragments, paired mutations cannot be too far apart, as they need to fall within the same cluster to be incorporated into a single dsDNA fragment. The allowable size range of dsDNA fragments depends on the synthesis provider; by default, our settings follow the specifications recommended by IDT that range from 300 to 1500 bp. The gene of interest must be provided in FASTA format, and the plasmid sequence containing the gene of interest should be available in GenBank (.gb) or SnapGene (.dna) format. Values for dsDNA fragment lengths and price per base pair can be adjusted to match the specifications of your DNA supplier.

### 3.2 Output

For every mutant, a complete vector sequence file in GenBank and SnapGene format is generated that can readily be imported into a sequence manager such as Benchling (The Benchling development team, 2024) or SnapGene (The SnapGene development team, 2024). These vector sequences contain the mutation of interest as well as the cloning and sequencing primers. For ordering of the dsDNA fragments and primers, a complete list of sequences is generated to streamline the ordering process. The localization of the fragment regions as well as the mutations within the gene of interest is also visualized using the plotting library DnaFeaturesViewer to easily interpret the results (Zulkower and Rosser, 2020).

### 3.3 Validation with a random synthetic test set

To test PyVADesign and to estimate the limits of the tool regarding the maximum insert and deletion length and the distance between paired mutations, we generated a synthetic dataset. For this, we introduced five random point mutations along with an additional insertion, deletion, or paired mutation of a defined length. Specifically, we systematically increased the length of the insertion, deletion, or paired mutation from 1 to 600 in steps of 5, repeating the process for each length. This procedure was performed for three different genes and repeated 10 times. Next, we designed dsDNA fragment regions for each mutated plasmid using the default settings, based on the synthesis specifications of IDT, and calculated the percentage of cases where dsDNA fragments could be designed; see Table S1, available as supplementary data at Bioinformatics online. We found that dsDNA fragments could be designed for deletions up to 185 residues, inserts of 115 residues, and paired mutations within 230 residues with a success rate over 90%. This indicates that user-specified input mutations falling within these boundaries can typically be incorporated into a single fragment region and inserted using a single PCR. It is important to note that these constraints do not reflect analytical limitations of the PyVADesign algorithm itself. Rather, they stem from the practical synthesis limitations imposed by DNA fragment providers. Starting from a list of input mutations, the design of the dsDNA fragments and primer sets for opening up the plasmids and sequence validation takes approximately 2 minutes.

### 3.4 Experimental validation

To showcase the applicability of our approach, we designed 24 mutations in the DNA polymerase DnaE1, an essential protein of *Mycobacterium smegmatis*. The PyVADesign parameters, the fragment regions, the dsDNA fragments, and the associated primers can be found in Fig. 1 and Tables S2 and S3, available as supplementary data at Bioinformatics online. The 24 mutations contained 18 point mutations, four double mutations, and two deletions. The 24 mutations were distributed over three fragment regions. The dsDNA fragments were combined simultaneously with their appropriate target plasmid and transformed into DH5α cells. Using this approach, all 24 mutant plasmids were generated within a span of two weeks.

## 4 Conclusion

In this paper, we present a novel approach for the generation of large mutant libraries using commercially available dsDNA fragments in combination with an IVA cloning approach. We implemented a Python tool that automates the process of designing dsDNA fragments and associated primers. Size limits of the dsDNA fragments for inserts and deletions were evaluated, and the approach was experimentally validated.

In some cases, PyVADesign fails to identify a clustering solution for the input mutations due to the distances between them. For cases where paired mutations are too far apart for clustering but within the established limits, introducing intermediate (e.g. silent) mutations between the two most widely spaced mutations helps satisfy constraints by reducing intra-cluster distances and shifting the medoid location. When the distance between paired mutations exceeds the applicability domain, users can manually introduce one distant mutation into the plasmid and rerun PyVADesign from this pre-mutated plasmid to overcome this limitation. In future versions, support for multi-fragment assembly could further extend the method’s applicability domain. As an open-source tool with minimal user input requirements, PyVADesign is intended to be accessible to a diverse set of users. Additionally, by reducing the number of required PCRs, the tool enhances both the efficiency and scalability of mutant generation.

## Supplementary Material

btaf433_Supplementary_Data

## Data Availability

The source code is available at https://github.com/CDDLeiden/PyVADesign. All code and data are archived via Zenodo (https://doi.org/10.5281/zenodo.15057525).

## References

[btaf433-B1] Agarwal PK. Enzymes: an integrated view of structure, dynamics and function. Microbial Cell Factories 2006;5:2. 16409630 10.1186/1475-2859-5-2 PMC1379655

[btaf433-B2] Bachman J. 2013. Chapter ninteen—site-directed mutagenesis. In: Lorsch J. (ed), Methods in Enzymology, Laboratory Methods in Enzymology: DNA. Amsterdam, Netherlands: Academic Press, pp. 241–248. 10.1016/B978-0-12-418687-3.00019-7 24011050

[btaf433-B3] Forloni M , Liu AY, Wajapeyee N et al Methods for in vitro mutagenesis. Cold Spring Harb Protoc 2019;2019:749–55. 10.1101/pdb.top097733 31792145

[btaf433-B4] Froger A , Hall JE. Transformation of plasmid DNA into E. coli using the heat shock method. J Vis Exp 2007;253:253–4. 10.3791/253 PMC2557105 18997900

[btaf433-B5] García-Nafría J , Watson JF, Greger IH et al IVA cloning: a single-tube universal cloning system exploiting bacterial in vivo assembly. Sci Rep 2016;6:27459. 27264908 10.1038/srep27459 PMC4893743

[btaf433-B6] Hiraga K , Mejzlik P, Marcisin M et al Mutation maker, an open source oligo design platform for protein engineering. ACS Synth Biol 2021;10:357–70. 33433999 10.1021/acssynbio.0c00542

[btaf433-B7] Hughes RA , Ellington AD. Synthetic DNA synthesis and assembly: putting the synthetic in synthetic biology. Cold Spring Harb Perspect Biol 2017;9:a023812. 28049645 10.1101/cshperspect.a023812 PMC5204324

[btaf433-B8] Kaufman L , Rousseau P. 1990. Partitioning around medoids. In: Finding Groups in Data. Hoboken, New Jersey, USA: John Wiley & Sons, Ltd, pp. 68–125.

[btaf433-B9] Kunzmann P , Hamacher K. Biotite: a unifying open source computational biology framework in python. BMC Bioinformatics 2018;19:346. 30285630 10.1186/s12859-018-2367-z PMC6167853

[btaf433-B10] Leonte RC, Fischer PD, Herguedas B et al IVA Prime: automated primer design for in vivo assembly cloning. Nucleic Acids Res 2025;53:gkaf386. 10.1093/nar/gkaf386 PMC12230671 40322915

[btaf433-B11] Loewe L , Hill WG. The population genetics of mutations: good, bad and indifferent. Philos Trans R Soc Lond B Biol Sci 2010;365:1153–67. 20308090 10.1098/rstb.2009.0317 PMC2871823

[btaf433-B12] Morris R , Black KA, Stollar EJ et al Uncovering protein function: from classification to complexes. Essays Biochem 2022;66:255–85. 35946411 10.1042/EBC20200108 PMC9400073

[btaf433-B13] Rozen S , Skaletsky H. Primer3 on the WWW for general users and for biologist programmers. Methods Mol Biol 2000;132:365–86. 10547847 10.1385/1-59259-192-2:365

[btaf433-B14] Sayers EW , Bolton EE, Brister JR et al Database resources of the national center for biotechnology information. Nucleic Acids Res 2022;50:D20–D26. 34850941 10.1093/nar/gkab1112 PMC8728269

[btaf433-B15] Sellés Vidal L , Isalan M, Heap JT et al A primer to directed evolution: current methodologies and future directions. RSC Chem Biol 2023;4:271–91. 37034405 10.1039/d2cb00231k PMC10074555

[btaf433-B16] Sen N , Anishchenko I, Bordin N et al Characterizing and explaining the impact of disease-associated mutations in proteins without known structures or structural homologs. Brief Bioinform 2022;23:bbac187. 35641150 10.1093/bib/bbac187 PMC9294430

[btaf433-B17] Tait RC. The application of molecular biology. Curr Issues Mol Biol 1999;1:1–12. 11475693

[btaf433-B18] The Benchling development team 2024. Benchling.

[btaf433-B19] The scikit-learn-extra development team 2020. scikit-learn-extra: a Python module for machine learning that extends scikit-learn.

[btaf433-B20] The SnapGene development team 2024. SnapGene.

[btaf433-B21] Untergasser A , Cutcutache I, Koressaar T et al Primer3—new capabilities and interfaces. Nucleic Acids Res 2012;40:e115. 22730293 10.1093/nar/gks596 PMC3424584

[btaf433-B22] Weiner MP , Costa GL, Schoettlin W et al Site-directed mutagenesis of double-stranded DNA by the polymerase chain reaction. Gene 1994;151:119–23. 7828859 10.1016/0378-1119(94)90641-6

[btaf433-B23] Westermarck J , Ivaska J, Corthals GL et al Identification of protein interactions involved in cellular signaling. Mol Cell Proteomics 2013;12:1752–63. 23481661 10.1074/mcp.R113.027771 PMC3708163

[btaf433-B24] Zulkower V , Rosser S. DNA features viewer: a sequence annotation formatting and plotting library for python. Bioinformatics 2020;36:4350–2. 32637988 10.1093/bioinformatics/btaa213

