# Supplementary material for: PyVADesign: a python-based cloning tool for one-step generation of large mutant libraries
Source: Bioinformatics. 2025 Sep 1;41(9):btaf433. doi: 10.1093/bioinformatics/btaf433 (PMC12449254; doi:10.1093/bioinformatics/btaf433)
Supplement: btaf433_Supplementary_Data [file btaf433_supplementary_data.docx]

# *Supplementary Information*

**PyVADesign: a Python-based cloning tool for one-step generation of large mutant libraries**

R.C.M. Kuin^1,2^ , M.H. Lamers^2^, G.J.P. van Westen^1^

**Table 1.** Success rates on synthetic dataset. Success rates (50%, 75%, 90%) of designing dsDNA fragments on a synthetic dataset. For each length (with steps of five) we used 30 different random input mutations to estimate the limits of PyVADesign using the default parameters.

|  | Success >50% | Success >75% | Success >90% |
| --- | --- | --- | --- |
| Insertion length (bp) | 220 | 170 | 115 |
| Deletion length (bp) | 350 | 245 | 185 |
| Distance between paired mutations (bp) | 350 | 315 | 230 |

| Primer name | Direction | Primer set | Sequence (5’ to 3’) |
| --- | --- | --- | --- |
| fw_DNABlock-1 | Forward | Opening-up target plasmid | GGTGACGGCTACTACCT |
| rv_DNABlock-1 | Reverse | Opening-up target plasmid | CTCCACGCCGATGATCG |
| fw_DNABlock-2 | Forward | Opening-up target plasmid | CGCGCTGTACCGGCC |
| rv_DNABlock-2 | Reverse | Opening-up target plasmid | TCGATATCGATGTCGGGCAT |
| fw_DNABlock-3 | Forward | Opening-up target plasmid | CGATCTCTTCGGCAGCAATG |
| rv_DNABlock-3 | Reverse | Opening-up target plasmid | GCGGGATAGTTGGCCTTGAG |
| seq_fw_DNABlock-1 | Forward | Sequence validation | ACTGGCCTTGTGTTAAAAATGG |
| seq_rv_DNABlock-1 | Forward | Sequence validation | CACTTGTCGGCTGCGTA |
| seq_fw_DNABlock-2 | Forward | Sequence validation | TGACGGTCTGCTCGAGA |
| seq_rv_DNABlock-2 | Forward | Sequence validation | ATGTCTTGGCCGACCGA |
| seq_fw_DNABlock-3 | Forward | Sequence validation | ATGGACTTCCTGGGCCT |
| seq_rv_DNABlock-3 | Forward | Sequence validation | GCGCACTCACCTTGTCA |

**Table 2**. Primers used to linearize the expression plasmid and to validate dsDNA fragment insertion.

| Parameter name | Description | Value |
| --- | --- | --- |
| max_DNABlock_length | Maximum dsDNA fragment length | 1500 |
| min_DNABlock_length | Minimum dsDNA fragment length | 300 |
| min_overlap | Minimum overlap between dsDNA fragment and plasmid | 15 |
| amount_optimization | Selecting the lowest number of fragment regions | True |
| silent_mutations | Introducing silent mutations at the beginning and end of dsDNA fragment | False |

**Table 3**. PyVADesign parameters used for the generation of dsDNA fragments for experimental validation. The minimum length of the dsDNA fragments are based on the requirements of IDT.

**
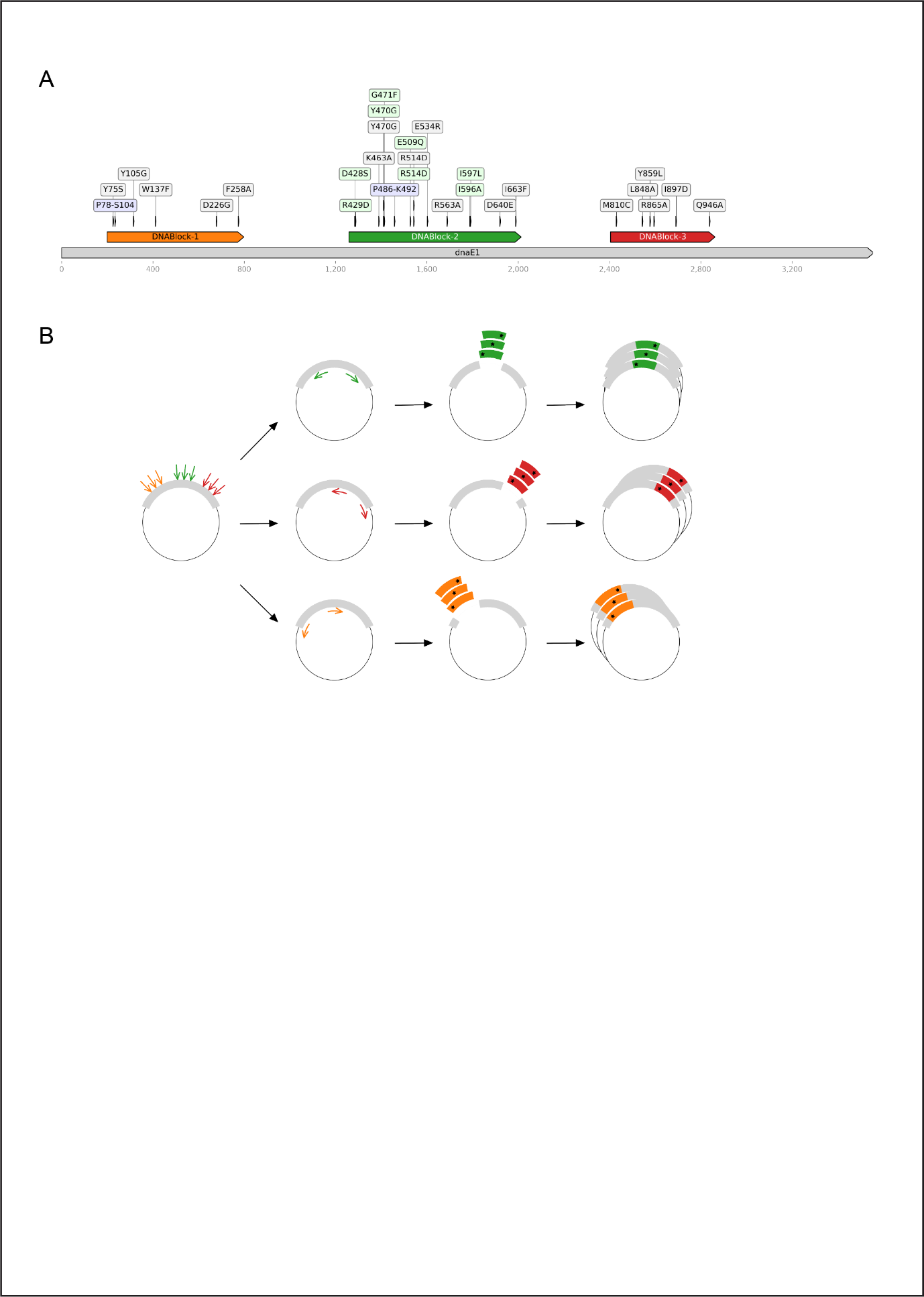
**

**Figure 1**. **(A)** Overview of the gene fragments corresponding to the selected mutations used for experimental validation. dsDNA fragments are visualized using DnaFeaturesViewer (Zulkower and Rosser 2020). Mutations are represented as follows: single point mutations in grey, paired point mutations in green, and inserts in blue. Each fragment region contains the following number of dsDNA fragments: orange – 6, red – 6, and green – 12. **(B)** Assembling scheme of the dsDNA fragments. The dsDNA fragments are assembled simultaneously into the target gene (grey) Each variant containing double mutations in DNABlock-2 was constructed by incorporating multiple mutations within a single dsDNA fragment, allowing the mutant plasmid with the double mutations to be generated in a single step.
